# Questionnaire of chronic illness care in primary care-psychometric properties and test-retest reliability

**DOI:** 10.1186/1472-6963-11-295

**Published:** 2011-11-02

**Authors:** Jost Steinhaeuser, Antje Miksch, Dominik Ose, Katharina Glassen, Iris Natanzon, Joachim Szecsenyi, Katja Goetz

**Affiliations:** 1 Department of General Practice and Health Services Research, University of Heidelberg, Voßstraße 2, 69115 Heidelberg, Germany

**Keywords:** Multimorbidity, Chronic Care, Health Care Delivery, ACIC, Chronic Care Model

## Abstract

**Background:**

The Chronic Care Model (CCM) is an evidence-based approach to improving the structure of care for chronically ill patients with multimorbidity. The Assessment of Chronic Illness Care (ACIC), an instrument commonly used in international research, includes all aspects of the CCM, but cannot be easily extended to the German context. A new instrument called the "Questionnaire of Chronic Illness Care in Primary Care" (QCPC) was developed for use in Germany for this reason. Here, we present the results of the psychometric properties and test-retest reliability of QCPC.

**Methods:**

A total of 109 family doctors from different German states participated in the validation study. Participating physicians completed the QCPC, which includes items concerning the CCM and practice structure, at baseline (T0) and 3 weeks later (T1). Internal consistency reliability and test-retest reliability were evaluated using Cronbach's alpha and Pearson's r, respectively.

**Results:**

The QCPC contains five elements of the CCM (decision support, delivery system design, self-management support, clinical information systems, and community linkages). All subscales demonstrated moderate internal consistency and moderate test-retest reliability over a three-week interval.

**Conclusions:**

The QCPC is an appropriate instrument to assess the structure of chronic illness care. Unlike the ACIC, the QCPC can be used by health care providers without CCM training. The QCPC can detect the actual state of care as well as areas for improvement of care according to the CCM.

## Background

The number of patients with multiple chronic diseases is growing world-wide [1]. Multimorbidity affects the quality of life, utilization of health care services and mortality [2]. In general practice, multimorbidity is defined as the co-occurrence of two or more chronic conditions [3]. Little is known about the optimal management of patients with multimorbidity and interactions between multiple drugs and diseases within the framework of chronic illness care.

The Chronic Care Model (CCM) is an evidence-based approach to improving care for chronically ill patients with multimorbidity [4]. It was developed in the United States during the 1990s [5]. The Assessment of Chronic Illness Care (ACIC) is internationally used to determine whether and to what extent an implemented system is optimizing the quality of care according to the CCM [6,7].

The ACIC assesses all six elements of the CCM (organization of the healthcare delivery system, decision support, delivery system design, self-management support, clinical information system, and community linkages). To measure how care is provided in German practices, researchers have started to translate-and tried to culturally adapt the ACIC instrument. During this process, we determined that the ACIC is not fully applicable to the German health care system. These limitations are due to a multifaceted understanding of terms, different levels of knowledge of the CCM, and fundamental differences between health care systems [8]. The CCM has proven benefits for chronically ill patients [9], and its implementation in primary care seems to be promising [10]. Therefore, a new instrument--the "Questionnaire of Chronic Illness Care in Primary Care" (QCPC)--was created by German researchers to measure the current status of care according to the CCM and potentials for improvement and further development. The QCPC includes the theoretical background of the CCM. Unlike using the ACIC, instrument, a physician in charge of a practice should be able to complete the QCPC without any special training in CCM. Here, we report the results of the psychometric properties and test-retest reliability of the QCPC.

## Methods

In a first step, we collected data on the CCM and factors influencing the care of patients in Germany with multiple chronic conditions based on a literature search and our own hypotheses. The ACIC instrument and questionnaires such as Patient Assessment of Chronic Illness Care (PACIC) [11] and the Commonwealth Fund survey [12] were assessed. Based on the theory behind these questionnaires, new items were developed according to the rule to design a questionnaire study in health care [13]. Additional topics were collected and items drafted in a qualitative process of brainstorming and consensus [8]. Most additions concerned the structure of practices as former research has shown that factors such as practice size rather than chronic care organization determine outcomes [14-16].

Nine general practitioners were asked to evaluate the questionnaire to determine whether each item was understandable, whether the content of an item was important for care of chronically ill patients, and whether any important aspects were missing. Details of this process are published elsewhere [8].

### Recruitment

A total of 288 general practitioners throughout Germany, each of which had participated in the European Practice Assessment for health care quality improvement [17] during the last two years, were invited by fax to participate in the validation study. One hundred nine general practitioners completed the questionnaire at baseline (T0 = test), and 87 completed it again 3 weeks later (T1 = retest). Therefore, a total of 87 data sets were available for reliability analysis.

### Questionnaire

The version of the QCPC used in the validation study contained 42 questions concerning care based on the CCM and 28 questions based on other hypotheses (23 regarding aspects of practice structure, 3 concerning quality management aspects, and 2 concerning disease management programs). The questionnaire is shown in the additional file 1. Five essential elements of the CCM (decision support, delivery system design, self-management support, clinical information systems and community linkages) were handled as subscales and analyzed in detail. Our previous experience has shown that the ACIC domain *healthcare delivery system *cannot be easily extended to the German context due to fundamental differences between health systems [8]. Therefore we did not included questions about this domain in the QCPC.

The "decision support" subscale consists of two items rated on a five-point Likert scale (1 = always to 5 = never). The "delivery system design" subscale consists of eight items, two of which are rated on a five-point Likert scale (1 = always to 5 = never), and the other six on a different five-point Likert scale (1 = 0% to 5 = more than 75%). The "self-management support" subscale consists of eight items rated on a five-point Likert scale (1 = always to 5 = never). The "clinical information systems" subscale comprises 15 items answered with a "1 = yes" or "2 = no". The "community linkages" subscale consists of nine items rated on a five-point Likert scale (1 = very satisfied to 5 = very unsatisfied).

### Statistical analysis

All analyses were carried out using SPSS 18.0 software (SPSS Inc., Chicago, IL USA). A hierarchical cluster analysis was performed to identify differences and similarities in the hierarchy structure of the different practices. Internal consistency was assessed using Cronbach's alpha, which indicates whether an item of a scale is appropriate for assessing the underlying concept of the scale [18]. Values for Cronbach's alpha range from 0 to 1. The closer they are to 0, the less related the items are to one another. Values above 0.60 are generally considered to indicate satisfactory internal consistency, and those above 0.80 indicate high internal consistency.

Test-retest reliability was assessed using the nonparametric Spearman rank order correlation coefficient (*r*) to determine the stability of the questionnaire. This criterion refers to the likelihood that a test will yield the same description of a phenomenon if the test is repeated and the phenomenon is unchanged [19]. Retest reliability is defined as correlation between the two test ratings. Spearman rank was computed as recommended in the original study. Spearman rank scores range from -1 to 1, where a score of 1 indicates the highest correspondence. QCPC items showing *r *values larger than 0.50 are considered to be very reliable. However, their reliability also depends on the expected stability of the investigated construct.

The nonparametric Wilcoxon matched paired test was used to test for differences between T0 and T1. If no significant differences were detected, the stability of the construct could be assumed. The level of significance was p < 0.05 respectively p < 0.01.

### Ethical approval

The study was fully approved by the ethics committee of the Medical Faculty of the University of Heidelberg (approval number S-090/2009).

## Results

Of 288 practices invited to participate in the validation study, the completed questionnaire was returned by 109 (38%) at T0 and by 87 (30%) at T1. The characteristics of our study participants are presented in Table 1. Cluster analysis revealed five distinct clusters, one of which contained 75 of the practices. This indicates that there was little overall variation.

**Table 1:** Characteristics of study participants

Female, n (%)	74 (67.9)
Male, n (%)	34 (31.2)

Age mean; (SD*)	54.0 (7.5)

Years of practice experience mean; (SD*)	18.56 (8.55)

Single-handed practice, n (%)	57 (52.3)

Group practice, n (%)	46 (42.1)

Inhabitants in practice area	

Less than 5,000, n (%)	17 (15.6)

5,000 to 20,000, n (%)	36 (33.0)

> 20,000 to 100,000, n (%)	20 (18.3)

More than 100,000, n (%)	36 (33.0)

* Standard deviation

Table 2 shows the results for the "decision support" and "delivery system design" subscales. The internal consistency (α) of the "decision support" subscale was determined to be 0.74. The correlation coefficients used for test-retest reliability were 0.70 and 0.66, respectively. Matched paired tests showed no significant differences. Internal consistency of the "delivery system design" subscale was 0.45. The correlation coefficients for test-retest reliability ranged from 0.48 to 0.67. Significant differences in one item of this subscale ("Are you informed when your patients receive hospital treatment") were detected, as reflected by p-values of 0.03. There were no significant differences in the other seven items of this subscale.

**Table 2:** Decision support and delivery system design subscale results

Use of evidence-based guidelines by patients with:*	T0 (n = 109) mean (SD)	T_x1 _(n = 85) mean (SD)	T_x2 _(n = 85) mean (SD)	**Wilcoxon matched pair test, p-value**^a^	Test-retest reliability: Spearman rho	
					**r**	**p-value^b^**

Single diseases	2.39 (0.71)	2.43 (0.72)	2.39 (0.76)	0.60	0.58	< 0.01

Multiple diseases	2.50 (0.76)	2.50 (0.77)	2.42 (0.80)	0.21	0.63	< 0.01

Reasons for referral**						

Differential diagnostic/therapeutic reasons	4.03 (1.01)	3.99 (1.00)	3.79 (1.02)	0.09	0.53	< 0.01

Own feeling of uncertainty	2.11 (0.57)	2.12 (0.62)	2.15 (0.59)	0.73	0.57	< 0.01

Request of a specialist	2.07 (0.94)	2.04 (0.94)	2.16 (0.84)	0.13	0.62	< 0.01

Disease management programs or guidelines	2.23 (0.92)	2.22 (0.90)	2.30 (0.95)	0.52	0.48	< 0.01

Patient request	2.79 (0.88)	2.84 (0.91)	2.78 (0.93)	0.49	0.62	< 0.01

Request by patient's family member	1.95 (0.64)	1.95 (0.65)	1.94 (0.67)	1.00	0.58	< 0.01

Do you know when your patients are being treated or admitted to hospital? *	2.12 (0.60)	2.11 (0.64)	2.25 (0.62)	0.03	0.52	< 0.01

How often does the provisional discharge letter provide all relevant information needed for continued care?*	2.24 (0.61)	2.24 (0.65)	2.24 (0.55)	1.00	0.67	< 0.01

* Five-point scale ranging from 1 "always" to 5 "never"

** Five point scale ranging from 1 "0%" to 5 "more than 75%"

^a ^Statistical significance of differences: P ≤ 0.05; ^b ^Statistical significance of differences: P ≤ 0.01

T_x1_, T_x2_, measurement points for re-test

Table 3 shows the results for the "self-management support" subscale. Internal consistency was for this subscale was 0.63, and the correlation of single items ranged from 0.50 to 0.67. The matched paired test showed in one item a significant differences (p = 0.02) which was called the assessment of drug history.

**Table 3:** Self-management support

Items*	T0 (n = 109) mean (SD)	T_x1 _(n = 87) mean (SD)	T_x2 _(n = 87) mean (SD)	**Wilcoxon matched pair test, p-value**^a^	Test-retest reliability: Spearman rho	
					**r**	**p-value^b^**

Handout of non individualized information sheets dealing with the disease on a daily basis and in difficult conditions	3.23 (0.94)	3.25 (0.95)	3.21 (0.89)	0.66	0.51	< 0.01

Handout of an individualized treatment plan with information on how to deal with the disease on an every day basis and in difficult conditions	2.91 (1.12)	3.09 (1.10)	2.96 (1.04)	0.26	0.54	< 0.01

Discussion of care/therapy options with the patients to achieve an agreed therapy concept	1.69 (0.63)	1.71 (0.65)	1.79 (0.67)	0.23	0.56	< 0.01

Assessment of drug history including OTC and prescription drugs from other physicians	1.49 (0.67)	1.49 (0.70)	1.64 (0.67)	0.02	0.63	< 0.01

Usage of specific instruments to calculate individual risks, e.g., for coronary heart disease	3.62 (1.07)	3.67 (1.04)	3.67 (1.04)	0.89	0.62	< 0.01

Handout of a patient-booklets for documentation, e.g., of blood glucose or pain levels	2.26 (0.85)	2.38 (0.85)	2.35 (0.91)	0.78	0.65	< 0.01

Handout of guideline information	3.19 (1.02)	3.24 (1.04)	3.11 (0.90)	0.16	0.67	< 0.01

Involvement of patient family members, if desired	1.75 (0.77)	1.80 (0.80)	1.86 (0.80)	0.48	0.50	< 0.01

* Five-point scale ranging from 1 "always" to 5 "never"

^a ^Statistical significance of differences: P ≤ 0.05; ^b ^Statistical significance of differences: P ≤ 0.01

The results for the "clinical information systems" subscale are presented in Table 4. Internal consistency for this subscale was 0.68. The correlation values determined as test-retest reliability ranged from 0.29 to 0.98, and no significant differences between items were found.

**Table 4:** Clinical information systems

Items*	T0 (n = 109) mean (SD)	T_x1 _(n = 85) mean (SD)	T_x2 _(n = 85) mean (SD)	**Wilcoxon matched pair test, p-value**^a^	Test-retest reliability: Spearman rho	
					**r**	**p-value**^b^

Access to internet	1.23 (0.42)	1.26 (0.44)	1.25 (0.43)	0.66	0.85	< 0.01

E-mail contact with colleagues	1.33 (0.47)	1.34 (0.48)	1.37 (0.49)	0.48	0.77	< 0.01

E-mail contact with patients	1.56 (0.50)	1.60 (0.49)	1.56 (0.50)	0.32	0.76	< 0.01

Access to medical literature	1.07 (0.25)	1.08 (0.28)	1.08 (0.28)	1.00	0.58	< 0.01

Access to guidelines	1.10 (0.30)	1.12 (0.33)	1.14 (0.35)	0.71	0.65	< 0.01

Internet homepage	1.54 (0.50)	1.58 (0.50)	1.56 (0.50)	0.32	0.98	< 0.01

Brochure with practice information	1.44 (0.50)	1.41 (0.50)	1.38 (0.52)	0.48	0.81	< 0.01

Flyer for therapeutic/diagnostic options	1.36 (0.48)	1.27 (0.45)	1.27 (0.45)	1.00	0.66	< 0.01

Appointment scheduling	1.47 (0.50)	1.51 (0.50)	1.51 (0.50)	1.00	0.95	< 0.01

Documentation/patient records	1.05 (0.21)	1.05 (0.23)	1.03 (0.16)	0.32	0.29	0.01

Reminders	1.26 (0.44)	1.25 (0.43)	1.26 (0.44)	0.74	0.70	< 0.01

Checking for interactions when prescribing drugs	1.24 (0.43)	1.26 (0.44)	1.32 (0.47)	0.32	0.46	< 0.01

Access to data from hospitals	1.86 (0.37)	1.90 (0.34)	1.96 (0.46)	0.44	0.41	< 0.01

Screening for patients	1.19 (0.39)	1.21 (0.41)	1.19 (0.40)	0.74	0.61	< 0.01

Patient lists with appointments	1.37 (0.48)	1.36 (0.48)	1.36 (0.48)	1.00	0.57	< 0.01

* Two-point scale ranging from "1" (yes) to "2" (no)

^a ^Statistical significance of differences: P ≤ 0.05; ^b ^Statistical significance of differences: P ≤ 0.01

Table 5 shows the results for the "community linkages" subscale. Internal consistency was 0.78, and the correlation coefficients for test-retest reliability ranged from 0.50 to 0.62. One item of this subscale, "satisfaction with other GP practices", showed a significant differences (p = 0.04). No significant differences in the other eight items of this subscale were detected.

**Table 5:** Community linkages

Satisfaction with the exchange of information with:*	T0 (n = 109) mean (SD)	T_x1 _(n = 85) mean (SD)	T_x2 _(n = 85) mean (SD)	Wilcoxon matched pair test, p-value^a^	Test-retest reliability: Spearman rho	
					**r**	**p-value**^b^

Other GP practices	2.35 (0.94)	2.39 (0.98)	2.20 (0.95)	0.04	0.62	< 0.01

Specialist practices	2.31 (0.77)	2.35 (0.84)	2.33 (0.72)	0.75	0.62	< 0.01

Psychotherapists	3.13 (0.95)	3.02 (0.98)	3.09 (1.03)	0.67	0.60	< 0.01

Physiotherapists/occupational therapists	2.95 (0.83)	2.59 (0.82)	2.67 (0.89)	0.32	0.52	< 0.01

Nursing homes	2.46 (0.86)	2.50 (0.89)	2.60 (0.95)	0.31	0.55	< 0.01

Medical supply stores	2.80 (0.87)	2.77 (0.85)	2.83 (0.89)	0.47	0.62	< 0.01

Pharmacies	2.14 (0.95)	2.09 (0.92)	2.13 (0.97)	0.75	0.59	< 0.01

Hospitals	2.50 (0.85)	2.50 (0.81)	2.57 (0.77)	0.35	0.58	< 0.01

Rehabilitation facilities	2.57 (0.86)	2.56 (0.83)	2.55 (0.83)	0.87	0.50	< 0.01

* Five-point scale ranging from 1 "very satisfied" to 5 "very unsatisfied"

^a ^Statistical significance of differences: P ≤ 0.05; ^b ^Statistical significance of differences: P ≤ 0.01

T_x1_, T_x2_, measurement points for the re-test

Because one item of the "community linkages" subscale ("satisfaction with the exchange of information with other GP practices") was not stable, it was deleted from the questionnaire.

## Discussion

This report describes the results of the psychometric properties and test-retest reliability of the QCPC, which contains elements of the CCM. The results indicate that the QCPC might be the first instrument giving psychometric properties insight into the question of whether and to what degree German practices have already implemented CCM elements into daily care of patients with one or more chronic conditions. The importance of practice structure has been demonstrated in previous research [16,20]. Almost two-thirds of our participating practices belong to one cluster and shows that a small inter-practice variability regarding measure of quality of care exists. This is an important aspect for caring patients with chronic conditions at primary care [21]. However, more then one-third of the items on the QCPC relate to aspects of practice structure. The QCPC does not contain items relating to the CCM element "health system/organization of health care" because of its limited applicability in the German context. Knowledge transfer from one country to another has known limitations [22]. Therefore a country specific process has to be intended in any case of knowledge transfer. However, QCPC does contain the other five elements of the CCM (decision support, delivery system design, self-management support, clinical information systems and community linkages). All of these subscales demonstrated moderate internal consistency and moderate test-retest reliability over the three-week test-retest interval.

For "delivery system design" the internal consistency was low. One reason for this result might be that especially reasons for referral are very little reflected in daily practice. Stability, as assessed using the Wilcoxon matched paired test, was very good. Because one item of the "community linkages" subscale was not stable, it was deleted from the questionnaire. A low test-retest reliability was observed for the item "disease management programs or guidelines" (r = 0.48). The implementation of guidelines in German primary care practice is basic yet. Decision making is rather a matter of former individual hospital pathways but evidence [23]. Therefore treatment is due to individual behavior in each reason for encounter and might explain the low reliability score. We also found a low test-retest reliability of two items of the "clinical information systems" subscale; namely, "documentation and patient files" (r = 0.29) and "access to data from hospitals" (r = 0.41). Because it currently is not possible to access data directly from German hospitals, we decided to delete that item. Conversely, we decided that "documentation and patient files" was important for German physicians in terms of billing by the German Association of Statutory Health Insurance Physicians. Overall, the results of our study showed moderate to low values for test-retest reliability, which is related to the lower variance of some items [24]. However results need to be discussed in the light of the low response rate. This might be partly due to little interest in redesigning care and the length of the questionnaire.

### Strengths and weaknesses

The QCPC is a questionnaire for assessment of care according to the CCM with psychometric properties and test-retest reliability. The QCPC can be used in a large number of practices without previous teaching in CCM background. However, the construct of the questionnaire especially different measurement scales in one questionnaire does not allow exploring a factor analysis. To conduct an exploratory or confirmatory factor analysis the measurement scales should be consistent [25]. This specific problem is already known for the PACIC validation [26]. Further methodological research should determine the underlying factor structure through implementation of construct validity. Due to the study design a non-responder characteristic was not determined.

As the QCPC was designed for use by primary care physicians, we were unable to assess different points of view from other practice team members. Another limitation is that the preliminary QCPC results will not be comparable to those of past international studies.

## Conclusions

The QCPC is a questionnaire for assessment of care according to the CCM in Germany which was tested of their psychometric properties. Unlike the ACIC instrument, the QCPC can be used in a high number of practices as the respondents need no prior training in CCM. The QCPC can gauge the current state of care as well as areas for improvement of care. The ESTHER-cohort was the first study performed in the federal state of Saarland, Germany, in which the QCPC was used to survey whether structured care according to the CCM leads to better care of multimorbid (and frail) patients. The ESTHER-cohort includes almost 700 GPs and about 10,000 patients [27,28]. Outcome data from this study will make it possible to complete the next validation step.

## Competing interests

The authors declare that they have no competing interests.

## Authors' contributions

All authors made substantive contributions to the study. JS, AM, DO, KGl, IN, JSz, and KG contributed in producing the QCPC. JS drafted the manuscript and analysed together with KG the data. All authors made contributions to the manuscript. All authors have read and approved the final manuscript.

## Pre-publication history

The pre-publication history for this paper can be accessed here:

http://www.biomedcentral.com/1472-6963/11/295/prepub

## Supplementary Material

Additional file 1 **The Questionnaire of Chronic Illness Care in Primary Care (QCPC)**. English translation of the Questionnaire of Chronic Illness Care in Primary Care (QCPC). Click here for file
