# Supplementary material for: Questionnaire of chronic illness care in primary care-psychometric properties and test-retest reliability
Source: BMC Health Serv Res. 2011 Nov 2;11:295. doi: 10.1186/1472-6963-11-295 (PMC3339331; doi:10.1186/1472-6963-11-295)
Supplement: Additional file 1 — The Questionnaire of Chronic Illness Care in Primary Care (QCPC). English translation of the Questionnaire of Chronic Illness Care in Primary Care (QCPC). [file 1472-6963-11-295-S1.DOCX]

**Questionnaire of Chronic Illness Care in Primary Care**

**„QCPC“**

Please pay attention to the following instructions when filling in the form:

In this questionnaire a **chronic disease** is considered to be a disease that exists since three months and leads to functional and / or bio-psycho-social limitations.

The term **multimorbidity** indicates that a person has an additional chronic or acute disease.

| - *Please use only blue or black ink pen.* - *Please print written answers.* - *Please mark you checkbox clearly:* ⌧ - *If you marked a check box by mistake, please fill it in completely:* ◼ *and mark your right choice clearly:* ⌧ |
| --- |

By filling in this questionnaire you agree, that your statements can be used for research purposes. Data will be pseudonymous and handled confidentially. No third party will have access to data.

**Thank you for participating!**

**General information**

| 1. What year were you born in? Please indicate the year. | | | | | | | |
| --- | --- | --- | --- | --- | --- | --- | --- |
|  | |  |  | | |  |  |
| 2. Your sex? | |  | female | | |  | male |
| 3. How many years have you been working as a general practitioner (since finishing vocational training)? Please round up / down.  | | | | | | years | |
| 4. How many inhabitants live in the town/city in which your practice is located? | | | | | | | |
|  | < 5000 habitants | | |  | 5.000 – 20.000 habitants | | |
|  | > 20.000 - 100.000 habitants | | |  | > 100.000 habitants | | |
| 5. What field did you specialize in? | | | | | | | |
|  | non | | |  | General practitioner / family physician | | |
|  | Internal medicine | | |  | other: ______________________ | | |
| 6. What kind of practice do you work in? | | | | | | | |
|  | Single handed | | |  | Group practice | | |
|  | others: ______________________ | | |  |  | | |
| 7. Which additional qualifications do you or other physicians in your practice have? | | | | | | | |
| 1. | ___________________________________________________________________________ | | | | | | |
| 2. | ___________________________________________________________________________ | | | | | | |
| 3. | ___________________________________________________________________________ | | | | | | |
| 8. About how many patients do you take care of in a quarter (three months)? | | | | | | | |
|  | < 500 patients | | |  | > 500-1000 patients | | |
|  | > 1000-1500 patients | | |  | > 1500-2000 patients | | |
|  | > 2000 patients | | |  |  | | |
|  | | | | | | | |

| 9. Please indicate the percentage of patients you treat in your practice on average each quarter in each of the following samples. Please estimate. | | | | | | | | | | |
| --- | --- | --- | --- | --- | --- | --- | --- | --- | --- | --- |
| Patients over 60 years | | |  | | % | |  | |  |  |
| Patients with at least two chronic diseases | | |  | | % | |  | |  |  |
| Bed-ridden patients | | |  | | % | |  | |  |  |
| Patients you visit at home regularly | | |  | | % | |  | |  |  |
| Patients with a psychiatric disease (e.g.depression) | | |  | | % | |  | |  |  |
| Patients with migrational background | | |  | | % | |  | |  |  |
| 10. Where do you take care of your multimorbid patients? Please indicate percentage. | | | | | | | | | | |
| At home | %  | Nursing  home | | %  | | in GP %  | | | | |
| 11. How many palliative patients do you take care of during three months in average? | | | | | | | | | | |
|  | patients |  | |  | |  | |  | | |
| 12. How many patients with dementia do you take care of during three months in average? | | | | | | | | | | |
|  | patients | | |  | |  | |  | | |
| 13. How many percent of your daily workload do you spend with the following activities? Please estimate. | | | | | | | | | | |
| Counselling chronically ill patients | | |  | | % | |  | |  |  |
| Counselling family members of chronically ill patients | | |  | | % | |  | |  |  |
| Home visits | | |  | | % | |  | |  |  |
| Documentation / paper work | | |  | | % | |  | |  |  |
| Contact with other health care providers | | |  | | % | |  | |  |  |

| 14. Please indicate the number of staff working in your practice (including yourself)?  e.g. two half time staff would be one full time equivalent (1,00). | | | | | | | | | | | | | | | | |
| --- | --- | --- | --- | --- | --- | --- | --- | --- | --- | --- | --- | --- | --- | --- | --- | --- |
| Physician | | | | | | |  | | | | full time equivalent | | | |  , | |
| resident | | | | | | |  | | | | full time equivalent | | | |  , | |
| doctors’ assistant | | | | | | |  | | | | full time equivalent | | | |  , | |
| doctors’ assistant trainee | | | | | | |  | | | | full time equivalent | | | |  , | |
| nurse | | | | | | |  | | | | full time equivalent | | | |  , | |
| other: ________________________ | | | | | | |  | | | | full time equivalent | | | |  , | |
| 15. Does your practice participate in a GP-centered model? | | | | | | | | | | | | | | | | |
|  | Yes, since | |  | | |  | | | | No | | |  | | | intended |
| 16. Does your practice provide complementary medicine? | | | | | | | | | | | | | | | | |
|  | Yes | | |  | | | | | | | | No | | | | |
| 17. Does your practice provide services that are not covered by patient’s insurance? Patients are obliged to pay themselves. | | | | | | | | | | | | | | | | |
|  | Yes | | |  | | | | | | | | No | | | | |
| 18. Which of the diagnostic / therapeutic devices listed below do you have in your  practice? Multiple answers allowed. | | | | | | | | | | | | | | | | |
|  | ECG | | |  | | | | Ultrasound | | | | | | | | |
|  | Ergometry | | |  | | | | Ultrasound with a Doppler function | | | | | | | | |
|  | Spirometry | | |  | | | | Pocket Doppler | | | | | | | | |
|  | Defibrillator | | |  | | | | Proctoscope | | | | | | | | |
|  | Others : ______________________________________________________________ | | | | | | | | | | | | | | | |
| 19. How many hours do you work per week? | | | | | | | | | | | | | | | | |
|  | | hours per week | | |  | | | |  | | | | |  | | |
| 20. How many weeks of vacation do you have per year? | | | | | | | | | | | | | | | | |
|  | | weeks per year | | |  | | | |  | | | | |  | | |

Technical support

| 21. Do you have internet access in your practice? | | | | | | | | | | |
| --- | --- | --- | --- | --- | --- | --- | --- | --- | --- | --- |
|  | Yes | | |  | | No | | | | |
| 22. Do you use the internet for one of the following professional reasons …? | | | | | | | | | | |
| E-mail contact with colleagues | | |  | | Yes | | |  | | No |
| E-mail contact with patients | | |  | | Yes | | |  | | No |
| Access to (medical) information or literature | | |  | | Yes | | |  | | No |
| Access to guidelines | | |  | | Yes | | |  | | No |
| other: _________________________________________________________________ | | | | | | | | | | |
| 23. Which means of public relation does your practice use? | | | | | | | | | | |
| Internet Homepage | |  | | | Yes | |  | | No | |
| Brochure with practice information | |  | | | Yes | |  | | No | |
| Flyer for specific therapeutical or  diagnostic options in your practice | |  | | | Yes | |  | | No | |
| others: _________________________________________________________________ | | | | | | | | | | |
| 24. Do you use your software program for …? | | | | | | | | | | |
| Appointment scheduling | | |  | | Yes | |  | | No | |
| Documentation / patient records | | |  | | Yes | |  | | No | |
| Reminders / recall function (e.g. for follow up) | | |  | | Yes | |  | | No | |
| Checking for interactions when prescribing drugs | | |  | | Yes | |  | | No | |
| Access to data from hospitals (e.g. letter of discharge) | | |  | | Yes | |  | | No | |
| Screening for patients with specific diagnosis or indication for a specific treatment | | |  | | Yes | |  | | No | |
| Creating patient lists with appointments for specific tests or preventative measures | | |  | | Yes | |  | | No | |
| Other : _________________________________________________________________ | | | | | | | | | | |

Disease Management Programms (DMP)

| 25. Does your practice participate in one of the following disease management programmes? | | | | | | | | |
| --- | --- | --- | --- | --- | --- | --- | --- | --- |
|  | No | | Yes,  since less than a year | | | | Yes,  since more than a year | |
| Diabetes mellitus type 1 |  | |  | | | |  | |
| Diabetes mellitus type 2 |  | |  | | | |  | |
| Coronary heart disease |  | |  | | | |  | |
| Asthma |  | |  | | | |  | |
| COPD |  | |  | | | |  | |
| Breast cancer |  | |  | | | |  | |
| If you answered all questions with “no”, please continue with question 27. | | | | | | | | |
| 26. If you do participate in a disease management program, how many of your patients with the respective diagnosis are enrolled in the program? | | | | | | | | |
|  | | < 25 % | | 25-50 % | 51-75 % | > 75 % | | Dont know |
| Diabetes mellitus type 1 | |  | |  |  |  | |  |
| Diabetes mellitus type 2 | |  | |  |  |  | |  |
| Coronary heart disease | |  | |  |  |  | |  |
| Asthma | |  | |  |  |  | |  |
| COPD | |  | |  |  |  | |  |

Doctors’ assistant / nurse

| 27. Is anyone among your non physician staff responsible for providing one of the following services for chronically ill patients? | | | | |
| --- | --- | --- | --- | --- |
| Patient education; e.g. about nutrition |  | Yes |  | No |
| Telephone monitoring |  | Yes |  | No |
| Regular updating of patient lists, e. g. by searching the practice software |  | Yes |  | No |
| others: ___________________________________________________________________ | | | | |

| 28. Which additional qualifications do doctor assistants / nurses in your practice have? | | |
| --- | --- | --- |
| 1. | _______________________________________________________________________ | |
| 2. | _______________________________________________________________________ | |
| 3. | _______________________________________________________________________ | |
| 29. Would you like to pass on more tasks to your doctors’ assistant / nurse? | | |
|  | | Yes, the following ones: ________________________________________________ |
|  | | No, because: ________________________________________________________ |

Self management support

| 30. How do you support your chronically ill patients in self management?  Please give only one response for each question, ranging from „always“ to „never“ | | | | | | |
| --- | --- | --- | --- | --- | --- | --- |
|  | always | | 🡲 | 🡲 | 🡲 | never |
| Handout of **non** individualized information sheets dealing with the disease on a daily basis and in difficult conditions. |  |  | |  |  |  |
| Handout of an individualized treatment plan with information on how to deal with the disease on an every day basis and in difficult conditions. |  |  | |  |  |  |
| Discussion of options of care / therapy options with the patient to achieve an agreed therapy concept. |  |  | |  |  |  |
| Assessment of drug history including OTC and prescription drugs from other physicians. |  |  | |  |  |  |
| Use of specific instruments to calculate individual risks, e.g. for coronary heart disease |  |  | |  |  |  |
| Handout of a patient-booklet for documentation, e.g., of blood glucose or pain levels. |  |  | |  |  |  |
| Handout of guideline information. |  |  | |  |  |  |
| Involvement of patient family members, if desired. |  |  | |  |  |  |

| 31. Do you support your chronically ill patients by refereeing them to one of the following services? | | | | |
| --- | --- | --- | --- | --- |
|  | **Yes, I do it by myself**  **respectively in cooperation with**  **someone** | **Yes, I provide information about** | **I would provide this, but no such offers exist nearby** | **No, I do not support this** |
| Self-help groups (support groups?) |  |  |  |  |
| Patient meetings |  |  |  |  |
| Patient education, e.g.  for diabetes or back pain |  |  |  |  |
| Counselling, e.g. nutrition |  |  |  |  |
| Sport programs, e.g. walking or coronary heart sport |  |  |  |  |
| Community programs, e.g. courses in an adult education center |  |  |  |  |
| Other :__________________ |  |  |  |  |
| Other :__________________ |  |  |  |  |

Coordination of care

| 32. Do your patients have problems getting an appointment with a specialist or psychotherapist in your area? | | | |
| --- | --- | --- | --- |
| When the patient calls himself? | |  | Yes, with following specialist/psychotherapist:  ______________________________________________ |
|  | |  | No |
| When you call yourself? | |  | Yes, with following specialist/psychotherapist:  ______________________________________________ |
|  | |  | No |
| 33. How often do you call yourself to arrange an appointment? | | | |
|  | times a day | |  |

| 34. Which are the specific reasons for you to write a referral (please indicate percent)? Please estimate for each line! You can rate more than 100%. | | | | | | | | | | |
| --- | --- | --- | --- | --- | --- | --- | --- | --- | --- | --- |
|  | | **0 %** | | **1-25 %** | | **>25-50 %** | | **>50-75 %** | | **>75 %** |
| Differential diagnostic /therapeutic reasons | |  | |  | |  | |  | |  |
| Own feeling of uncertainty | |  | |  | |  | |  | |  |
| Request of a specialist | |  | |  | |  | |  | |  |
| Relief of own drug budget | |  | |  | |  | |  | |  |
| Disease management programs /  guidelines | |  | |  | |  | |  | |  |
| patient request | |  | |  | |  | |  | |  |
| Request by patients family member | |  | |  | |  | |  | |  |
| Other: ___________________________ | |  | |  | |  | |  | |  |
| 35. How satisfied are you with the exchange of information with following health providers? Please give only one response for each question, ranging from “very satisfied” to “very unsatisfied“. | | | | | | | | | | |
|  | very satisfied | | **🡲** | | **🡲** | | **🡲** | | very unsatisfied | |
| Other GP practices |  | |  | |  | |  | |  | |
| Specialist practices |  | |  | |  | |  | |  | |
| Psychotherapists |  | |  | |  | |  | |  | |
| Physiotherapists / occupational therapists |  | |  | |  | |  | |  | |
| Nursing homes |  | |  | |  | |  | |  | |
| Medical supply stores |  | |  | |  | |  | |  | |
| Pharmacies |  | |  | |  | |  | |  | |
| Hospitals |  | |  | |  | |  | |  | |
| Rehabilitation facilities |  | |  | |  | |  | |  | |
| Other :___________________ |  | |  | |  | |  | |  | |
| Other :___________________ |  | |  | |  | |  | |  | |

| 36. Do you know when your patients are being treated or admitted to hospital? | | | | | | | | |
| --- | --- | --- | --- | --- | --- | --- | --- | --- |
|  | | always | **🡲** | | | **🡲** | **🡲** | never |
|  | |  |  | | |  |  |  |
| 37. How often does the **provisional** discharge letter provide all relevant  information needed for continued care? | | | | | | | | |
|  | | always | **🡲** | | | **🡲** | **🡲** | never |
|  | |  |  | | |  |  |  |
| 38. Does the **final** discharge letter provide additional information for you? | | | | | | | | |
|  | Yes | | |  | No | | | |
| 39. Is there a procedure in your practice to make sure that medical and diagnostic results arrive in the practice e.g. histological results? | | | | | | | | |
|  | Yes | | |  | No | | | |
| 40. Are the responsibilities clearly defined so that medical results, needed for the patient’s next appointment, will be available? | | | | | | | | |
|  | Yes | | |  | No | | | |

Quality management

| 41. Which quality management system do you use? Tick all that apply | | | |
| --- | --- | --- | --- |
|  | **planned** | **already done** | **certification accomplished** |
| QEP |  |  |  |
| DIN ISO |  |  |  |
| KTQ |  |  |  |
| EPA |  |  |  |
| Others:_____________________________ |  |  |  |

| 42. What do you do with quality reports / feedback reports, e. g. on Disease Management Programmes or your prescriptions? Tick all that apply. | | | | | | | | |
| --- | --- | --- | --- | --- | --- | --- | --- | --- |
|  | Are ignored | | |  | Are discussed in team meetings | | | |
|  | Are read quickly | | |  | Are used to improve workflow | | | |
|  | Are read carefully | | |  |  | | | |
| 43. Do you assess patient satisfaction in your practice? Tick all that apply | | | | | | | | |
|  | Yes, by using a questionnaire | | |  | Yes, in a different way | | | |
|  | Yes, by using a suggestion box | | |  | No | | | |
| 44. How often did you have a planned team meeting within the last 12 months? | | | | | | | | |
|  | Weekly | | |  | Less then once a quarter | | | |
|  | Monthly | | |  | Never | | | |
|  | Once every three months (Quarterly) | | |  |  | | | |
| 45. How often do you use existing evidence based guidelines, when treating patients with a single disease? | | | | | | | | |
|  | | always | **🡲** | | | **🡲** | **🡲** | never |
|  | |  |  | | |  |  |  |
| Which one most often? _______________________________________________________ | | | | | | | | |
| 46. How often do you use existing evidence based guidelines, when treating patients with multiple diseases (multimorbid patients)? | | | | | | | | |
|  | | always | **🡲** | | | **🡲** | **🡲** | never |
|  | |  |  | | |  |  |  |
| Which one most often? _______________________________________________________ | | | | | | | | |

Further education

| 47. Does your non-physican staffs attend further education on care of chronically ill patients at least once a year? | | | | | |
| --- | --- | --- | --- | --- | --- |
|  | Yes, all of them |  | Yes, some of them |  | No |

| 48. During the last 12 months: How often did you attend further education (excluding “quality circles”) on one of the following aspects of care for chronically ill patients? | | | | | | | | |
| --- | --- | --- | --- | --- | --- | --- | --- | --- |
| Medical issues | |  | | | | | Times in 12 months | |
| Organizational issues | |  | | | | | Times in 12 months | |
| Other: __________________ | |  | | | | | Times in 12 months | |
| 49. During the last 12 months: How often did you participate in “quality circles”? | | | | | | | | |
|  | | 0 | | 1-2 | | 3-4 | | > 4 |
|  | |  | |  | |  | |  |
| 50. Due to which criteria do you choose further education? Tick all that apply | | | | | | | | |
|  | Appointed time/date | |  | | Topic/Issue | | | |
|  | Incentives | |  | | Location | | | |
|  | Speaker | |  | | Amount of further education (qualification) points provided | | | |
|  | Others: ______________________ | |  | |  | | | |

**Thank you for participating!**
